# Retinal Microvascular Calibres and Body Fat Indices in Young Adults

**DOI:** 10.3390/ijerph23020219

**Published:** 2026-02-10

**Authors:** Yandisa Ntsilane, Asakhanya Moshani, Melikhaya M. Bukhali, Nthai E. Ramoshaba

**Affiliations:** Department of Human Biology, Faculty of Medicine and Health Sciences, Walter Sisulu University, Nelson Mandela Drive, Mthatha 5117, South Africa; 221021795@mywsu.ac.za (Y.N.); 221055533@mywsu.ac.za (A.M.); 221097937@mywsu.ac.za (M.M.B.)

**Keywords:** cardiovascular diseases, retinal microvasculature, body fat

## Abstract

**Highlights:**

**Public health relevance—How does this work relate to a public health issue?**

- This study links a major modifiable risk factor to an indicator of systemic vascular health by investigating the relationship between excess body fat and changes in retinal microvascular calibres.
- Retinal microvascular calibres are useful for detecting early cardio-metabolic risk associated with excess adiposity by providing a non-invasive window to systemic microvascular health.

**Public health significance—Why is this work of significance to public health?**

- Associating body fat to changes in retinal microvascular calibres can help to identify individuals at a higher risk of developing cardiovascular diseases before clinical signs can manifest.
- This research is crucial for developing preventative strategies aimed at young adults as early changes in the retinal microvascular structure may precede the development of hypertension, diabetes and cardiovascular diseases.

**Public health implications—What are the key implications or messages for practitioners, policy makers and/or researchers in public health?**

- The use of simple measures of body fat with retinal imaging could be employed by healthcare professionals to enhance early risk identification in primary care.
- These results could be used by policy makers to advocate for early-life obesity focused interventions to reduce cardiovascular diseases burden in the future.

**Abstract:**

Adverse microvascular complications are early markers of cardiovascular risk and precede the development of cardiovascular diseases. An increase in adiposity has been associated with changes in retinal microvascular calibre in a specific age group. However, this association is scarce in young adults. Therefore, this study aimed to investigate the relationship between neck circumference and body fat with retinal microvascular calibres in young adults. In our cross-sectional study, 330 students aged (18–30 years) participated. Anthropometric measurements were taken according to standard procedures. Retinal photographs were obtained with an Optomed retinal camera and analyzed with MONA-REVA vessel analysis software to obtain microvascular calibres such as central retinal arteriolar equivalent (CRAE), central venular equivalent (CRVE) and artery-to-venous ratio (AVR). In the stepwise multiple regression analysis, neck circumference (NC) was negatively associated with CRAE [adjusted R^2^ = 0.050; β = −0.122 (95% CI = −0.963; −0.067), *p* = 0.024], and body fat percentage was positively associated with CRVE [adjusted R^2^ = 0.060; β = 0.157 (95% CI = 0.072; 0.364), *p* = 0.004] adjusted for age, gender, body mass index, smoking, alcohol and mean arterial pressure. For the first time in young adults, an increase in upper body fat was associated with retinal arteriolar narrowing, while an increase in total body fat was associated with retinal venular widening.

## 1. Introduction

Cardiovascular diseases (CVDs) remain a public health concern and continue to be a major cause of morbidity and mortality worldwide, accounting for about 18 million deaths each year [1]. The burden of CVDs is exceptionally high in low- and middle-income countries where over three-quarters of CVDs mortality occurs [2]. There is growing evidence that the onset of CVDs may be traced to vascular, metabolic, and other processes that start in early life [3]. Therefore, identifying the main risk factors for CVDs has emerged as a subject of interest in clinical and public health sectors [4].

Adverse microvascular changes are early markers of cardiovascular risk and precedes the development of CVDs [5]. The retinal microvasculature exhibit similar morphological and physiological properties with other microvasculature such as the cerebral microvasculature [6]. In addition, retinal imaging has emerged as a unique tool that can be used to directly and noninvasively assess and monitor retinal microvasculature, providing a window into systemic microcirculation [7]. Previous studies have shown that alterations in the structure and function of retinal vessels, such as retinal arteriolar narrowing have been linked to high blood pressure, heart attack, and stroke [8], while retinal venular widening has been associated with systemic inflammation [9].

An increase in adiposity is known to induce microvascular complications [10]. Adiposity can be assessed using cheap and easy-to-use methods such as anthropometric indices, namely body mass index (BMI), waist circumference, neck circumference (NC), body fat percentage, and waist-to-height ratio, etc. However, NC and body fat are known to be good markers for assessing adiposity [11,12,13]. Only one study conducted by Tapp et al. 2020 has reported an association between body fat and changes in the retinal microvasculature, such as venular widening in adults aged 40 to 69 years [14]. However, the relationship between NC and body fat with retinal microvascular calibres is scarce in young adults. Therefore, this study aimed to investigate the association between NC and body fat with retinal microvascular calibres among young adults, which can provide valuable insights into the effect adiposity has on early microvascular structure, which can aid in the early detection and prevention of CVDs.

## 2. Materials and Methods

### 2.1. Study Design and Data Collection

This study employed a cross-sectional quantitative study design with a sample size of 330 students from Walter Sisulu University (WSU). This study was conducted in Nelson Mandela Drive Campus (NMD) located in Mthatha in the Eastern Cape Province, South Africa. To perform our multivariable regression analysis a G-power calculator was used and it showed that a minimum sample size of n = 89 would be required with an effect size of 0.15, alpha set to 0.05, and power to 0.95 [15,16].

### 2.2. Inclusion/Exclusion Criteria

Students from WSU aged 18–30 years without any history of cardiovascular, renal, and endocrine diseases were recruited to participate in this study. Furthermore, pregnant women were excluded from the study as well as those with eyes diseases such as diabetic retinopathy, hypertensive retinopathy, and glaucoma.

### 2.3. Recruitment

Participant were recruited by word of mouth from their respective residences and around WSU, as well as through posters and social media platforms to the physiology lab where the data was collected. The convenience sampling method was used; the participant were selected based on their availability and willingness to participate.

### 2.4. Data Collection

A general demographic and lifestyle questionnaire was filled by each participant with regard to age, gender, ethnicity, self-reported smoking, and self-reported alcohol consumption.

### 2.5. Measurements

#### 2.5.1. Anthropometric Measurements

Anthropometric measurements were performed by the researchers and well-trained assistants following the guidelines of the International Society for the Advancement of Kinanthropometry [17]. A SECA 213 portable stadiometerf (SECA, Hamburg, Germany) was used to measure height to the nearest 0.1 cm. The students were required to stand with their feet together and their heels, buttocks, and upper back against the scale. Then, they were instructed to maintain their head in the Frankfort plane while taking and holding a deep breath. A slight upward lift was applied via the mastoid processes. The base of the stadiometer was lowered to the vertex of the head, and a small pressure was put on to touch the top of the head if there was too much hair.

The neck circumference (NC) was obtained using a flexible tape (Lufkin Steel Tape, W606PM, Lufkin, TX, USA; Apex, NC, USA). The head of the participant was in a resting position whilst facing forward. The tape was placed in a horizontal plane around the neck above the thyroid cartilage. While measuring the tape was not pulled too tightly in order to avoid compressing the tissues around the area.

Body weight was obtained using an electronic scale (SECA, Hamburg, Germany) to the nearest 0.1 kg. Before, participants could get onto the scale, the readings were first checked, and the participants were instructed to remove their shoes and to stand on the centre of the scale without assistance and with their weight equally spread over on both feet. Their gaze had to be fixed to the front with their heads inclined upward. Then the readings were obtained.

Body fat percentage was measured using an electronic scale (SECA, Hamburg, Germany) before the participant could climb onto the scale they had to put their personal details such as age, gender, height and had to barefooted the scales uses bioelectrical impedance analysis in order to detect the body fat percentage. The BMI of the participant was obtained by dividing the weight in kilograms by the squared height in metres (BMI = kg/m^2^).

#### 2.5.2. Cardiovascular Measures

The clinical blood pressure (BP) was measured using an Omron M3 BP monitor (Omron, Kyoto, Japan). After resting for 3 to 5 min in a quiet room with a controlled room temperature, an appropriately sized cuff was fitted on the bare upper-left arm of the participants resting on the table with mid-arm at heart level, seated with the back supported by a chair legs uncrossed and feet flat on floor. Three readings of the systolic BP (SBP), diastolic BP (DPB), and heart rate (HR) were taken at 1 min interval. The average of the last two readings was calculated [18]. The mean arterial pressure was calculated as [DBP + 1/3(SBP − DBP)].

### 2.6. Retinal Vessel Analysis

Retinal blood vessel images focused on the optic disc (resolution of 1536 × 1536) of the participant’s right eye were captured with an Optomed Aurora portable digital retinal camera (Optomed Oy, Oulu, Finland) and were analyzed using the MONA-REVA vessel analysis software (version 2.1.1) developed by VITO as previously described [19,20]. A single trained operator from WSU was responsible for the vessel analysis while remaining blinded to the studies details to reduce analytical variability. A scale ratio was determined before the retinal images were analyzed. The distance between the centre of the macula (fovea) and the centre of the optic disc (blind spot) was measured to determine the scale ratio in pixels [21]. To obtain the scale ratio 4500 was divided by the distance (in pixels) between the macula and the blind spot in MONA REVA software. The resolution number was determined as 6.8 μm/pixel by averaging the individual resolution numbers from all retinal images. To obtain consistent retinal region across all fundus pictures in MONA-REVA, an annular region centred on the optic disc was defined with the inner and outer radii of the annulus set at 1.5 and 5 times the radius of the optic disc, respectively. Every vessel with a diameter larger than 100 µm laying in the perifoveal area were identified automatically and measured by the software. The image analysis algorithm based on a multiscale line filtering approach was then used to automatically segment the retinal vessels [22].Post-processing steps such as double thresholding, blob extraction, and removal of small, connected regions and filling of holes were perfomed. Using the MONA REVA vessel editing tool box, the calibres and labels of the retinal arterioles or retinal venules were corrected and verified. The revised Parr-Hubbard-Knudtson formula was used to measure the six largest arterioles and venules in order to obtain the retinal vascular calibres, which were then expressed as central retinal arteriolar equivalent (CRAE) and the central retinal venular equivalent (CRVE) [20]. The MONA-REVA software automatically calculated the arteriolar-to-venular ratio (AVR).

### 2.7. Statistical Analysis

To assess normal data distribution, formal tests [Kolmogorov–Smirnov test] and graphical methods [histograms and q-q plots] were used. Continuous data were reported as mean ± standard deviation. Categorical data was presented as frequencies and proportions (alcohol status and smoking status). Pearson correlation was used to investigate the associations between retinal parameters (CRAE, CRVE, and AVR) with NC and total body fat. In model 1, the stepwise multiple regression analysis was used to evaluate the independent associations between retinal microvascular parameters (CRAE, CRVE, and AVR) as dependent variables with NC as independent variable, with age, gender, smoking, alcohol, BMI, and MAP as covariates. In model 2, the stepwise multiple regression analysis was also used to evaluate the independent association between retinal microvascular parameters (CRAE, CRVE, and AVR) as dependent variables and total body fat as an independent variable with age, gender, smoking, alcohol, BMI, and MAP as covariates. The CRAE was also adjusted for CRVE and vice versa [23,24]. All the statistical analyses were performed using the Statistical Package for the Social Sciences (SPSS Inc., Chicago, IL, USA, version 26.0). The statistical significance was set at *p* < 0.05

## 3. Results

Table 1 below shows that the mean of age, body fat, NC, BMI, CRAE, CRVE, and AVR were 20.69 years; 32.00%; 33.24 cm, 24.73 kg/m^2^; 144.13 µm, 237.04 µm; 0.62, respectively.

**Figure 1:**
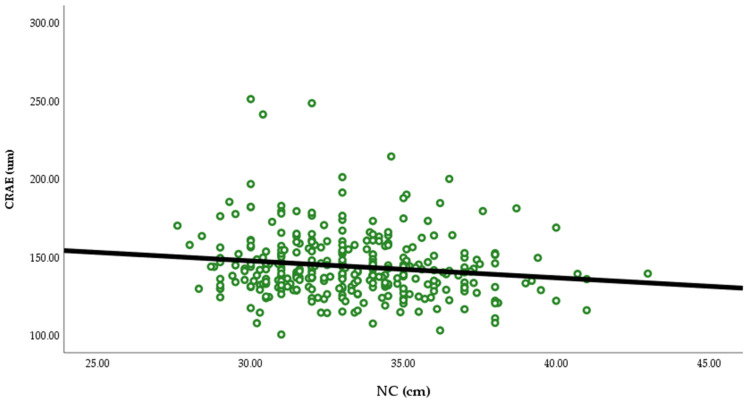
Illustration of the relationship between CRAE and NC.

**Figure 2:**
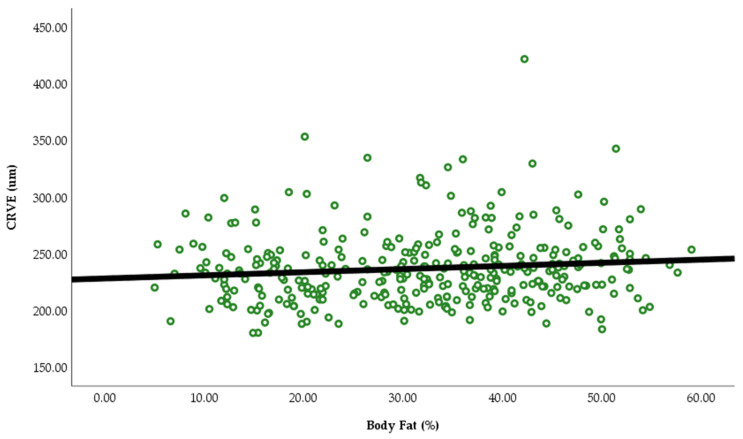
Illustration of the relationship between CRVE and body fat %.

In the Pearson correlation (Table 2, Figure 1 and Figure 2), NC was negatively associated with CRAE (r= −0.144, *p* = 0.009) and CRVE (r= −0.144, *p* = 0.039) and body fat was positively associated with CRVE (r = 0.162, *p* = 0.003). There was no association between NC and body fat with AVR.

In the stepwise multiple regression analysis (Table 3), NC maintained a consistent negative association with CRAE after adjusting for age, gender, BMI, CRVE, MAP, alcohol, and smoking. There was no association between NC with CRVE and AVR.

In the stepwise multiple regression analysis (Table 4), total body fat remained positively associated with CRVE after adjusting for age, gender, BMI, alcohol, smoking, and MAP. There was no association between total body fat with CRAE and AVR.

## 4. Discussion

This cross-sectional study investigated the relationship between NC and body fat with retinal microvascular calibres in young adults. Our findings revealed for the first time that NC is negatively associated with CRAE. In addition, body fat percentage was positively associated with CRVE. These findings suggest that an increase in upper body fat is linked to retinal arteriolar narrowing; moreover, an increase in body fat percentage relates to an increase in retinal venular widening. Therefore, an increase in overall adiposity may lead to early microvascular complications in young adults.

Studies linking retinal microvascular calibres with NC remain limited; however, a study by Lammert et al. 2012 discovered an association between NC and AVR in older participants, which is in contrast to our study [25]. The difference in results may be due to the relatively young and healthy status of our participants as well as variations in the measurement techniques. The mechanism that may link NC and CRAE has not yet been established. Nevertheless, we hypothesize that an increase in upper body subcutaneous fat measured by NC may function as an active endocrine organ that releases biochemical substances such as pro-inflammatory cytokines (e.g., tumour necrosis factor α, IL-6, leptin, fatty acid binding protein-4) [26,27]. TNF and IL-6 have been shown to induce endothelial dysfunction, a precursor of atherosclerosis and other CVDs leading to arteriolar narrowing [28]. However, more research is needed to define the mechanism linking body fat and retinal arteriolar narrowing in apparently healthy young adults.

The positive association between body fat percentage and CRVE found in our study is in alignment with a study conducted by Tapp et al. 2020 in adults aged 40–69 years, which also found an association between total fat percentage and wider venular diameter [14]. The possible mechanism that may link an increase in body fat percentage with CRVE might be explained by the fact that leptin, a mediator of long term regulation of energy balance, has been shown to be increased in individuals with excess body fat and is associated with venular widening [29]. Leptin leads to venular widening by altering the synthesis of nitric oxide [30].

Changes in retinal microvascular calibres are indicative of similar changes in other arterioles throughout the body, making it a window of CVDs [31]. The negative relationship between NC and CRAE suggests that as the NC increases by 1 unit, CRAE decreases by about 0.122 µm. Moreover, the positive relationship between body fat percentage and CRVE suggests that as body fat increases by 1 unit, CRVE increases about 0.157 µm. Therefore, these results may suggest that peripheral adiposity measured by NC and overall adiposity measured by body fat percentage are related to microvascular complications in young adults. This association may emphasize the importance of using non-invasive measures of body fat and retinal photography in clinical settings as screening tools in order to identify individuals at a higher risk of developing early vascular dysfunction, potentially leading to early interventions or prevention of CVDs.

The major strength of our study is the enrolment of young adults free of any known systemic diseases and the use of a validated technique to measure the retinal microvasculature and body fat. However, this study had limitations. Firstly, due to the study’s cross-sectional nature, causal inference cannot be drawn. Secondly, this study only included students from WSU as they are a relatively accessible and homogeneous population of young adults. However, the homogeneity of the population limits the generalizability of the findings; therefore, these results may not be representative of the broader population. Therefore, future longitudinal studies with larger and diverse populations should be explored to enhance the generalizability of findings and also to further investigate the link between adiposity measures and changes in retinal microvascular calibres over time. Furthermore, the use of convenience sampling methods introduces potential bias limiting the generalizability of the results. Lastly, even though we adjusted for multiple confounders in the regression analysis, data on lipid profile and physical activity, which may influence these findings, were not available for our study hence not included; however, we suggest future studies include these variables into their linear regression models in order to confirm our findings.

## 5. Conclusions

In young adults, an increase in upper body subcutaneous fat was associated with a retinal arteriolar narrowing, while an increase in overall adiposity was associated with retinal venular widening. These findings suggest that body fat may lead to early microvascular complications and can help to identify individuals at a higher risk of developing CVDs.

## Figures and Tables

**Table 1:** Characteristics of students.

Variables	Total (n = 330)
Age (years)	20.69 ± 2.34
Body Fat (%)	32.00 ± 12.63
NC (cm)	33.24 ± 2.71
BMI (kg/m^2^)	24.73 ± 5.44
Cardiovascular Measurements	
SBP (mmHg)	107.57 ± 11.97
DBP (mmHg)	68.97 ± 8.65
MAP (mmHg)	81.83 ± 8.46
HR (bpm)	78.30 ± 12.73
Retinal microvascular calibres	
CRAE (µm)	144.13 ± 20.31
CRVE (µm)	237.04 ± 31.51
AVR	0.62 ± 0.09
Lifestyle	
Alcohol, n (%)	133 (40.3)
Smoking, n (%)	57 (17.3)

Abbreviations: NC, neck circumference; BMI, body mass index, CRAE, central retinal arteriolar equivalent; CRVE, central retinal venular equivalent; AVR, artery-to-vein ratio; SBP, systolic blood pressure; DBP, diastolic blood pressure; HR, heart rate, MAP, mean arterial pressure.

**Table 2:** Pearson correlation between measures of body fat and retinal microvascular calibres.

Retinal Microvascular Calibres			
Variables	CRAE (µm)	CRVE (µm)	AVR
NC (cm)	r = −0.144 *p* = 0.009	r = −0.144 *p* = 0.039	r = −0.030 *p* = 0.585
Body Fat (%)	r = 0.024 *p* = 0.670	r = 0.162 *p* = 0.003	r = −0.096 *p* = 0.080

Abbreviations: NC, neck circumference; CRAE, central retinal arteriolar equivalent; CRVE, central retinal venular equivalent; AVR, artery to vein ratio.

**Table 3:** Stepwise multiple regression analysis between retinal microvascular calibres as dependent variables and NC as independent variables. Model 1.

	Retinal Microvascular Calibres	
	CRAE (µm) Adjusted R^2^ = 0.050	
Independent Variables	β (95% CI)	*p*-Value
NC (cm)	−0.122 (0.08; 0.29)	0.024
CRVE	0.189 (−0.08; 0.74)	0.001

Abbreviation: NC, neck circumference; CRAE, central retinal arteriolar equivalent; CRVE, central retinal venular equivalent. Age, gender, BMI, MAP, alcohol, and smoking were excluded during the stepwise regression.

**Table 4:** Stepwise multiple regression analysis between retinal microvascular calibres as the dependent variable and body fat as the independent variable. Model 2.

	Retinal Microvascular Calibres	
	CRVE (µm) Adjusted R^2^ = 0.060	
Independent Variables	β (95% CI)	*p*-Value
Body fat (%)	0.157 (0.07; 0.36)	0.004
CRAE	0.199 (0.10; 0.314)	<0.001

Abbreviation: CRAE, central retinal arteriolar equivalent; CRVE, central retinal venular equivalent. Age, gender, BMI, MAP, alcohol, and smoking were excluded during the stepwise regression.

## Data Availability

The data that supports the findings of this study are accessible from the corresponding author; however, access to this data is limited because it was used under authorization for the current study and hence, it is not publicly available.

## Abbreviations

The following abbreviations are used in this manuscript:

AVR	Artery-to-vein ratio
BP	Blood pressure
BMI	Body mass index
CVDs	Cardiovascular diseases
CRAE	Central retinal arteriolar equivalent
CRVE	Central retinal venular equivalent
MAP	Mean arterial pressure
NC	Neck circumference
WSU	Walter SisuluUniversity
